# Effectiveness of General Practitioners’ Involvement in Adult Vaccination Practices: A Systematic Review and Meta-Analysis of International Evidence

**DOI:** 10.3390/vaccines12121438

**Published:** 2024-12-20

**Authors:** Andrea Ceccarelli, Gabriele Munafò, Francesco Sintoni, Christian Cintori, Davide Gori, Marco Montalti

**Affiliations:** 1 Unit of Hygiene and Medical Statistics, Department of Biomedical and Neuromotor Sciences, University of Bologna, 40126 Bologna, Italy; 2 Forlì and Rubicone Health District, Romagna Local Health Authority, 47522 Cesena, Italy; 3 Sector of Collective Prevention and Public Health, Directorate General for Personal Care, Health, and Welfare, Emilia-Romagna Region, 40127 Bologna, Italy

**Keywords:** general practitioner, primary care, vaccine uptake, prevention departments, systematic review, effectiveness evaluation, vaccination campaigns

## Abstract

Background: General practitioners (GPs) and primary care units collaborate with Prevention Departments (PDs) to improve immunization by participating in vaccination campaigns, sharing tools, and implementing educational programs to raise patient awareness. This review aimed to identify effective strategies for involving GPs in PD vaccination practices. Methods: A systematic review following PRISMA guidelines was conducted on MEDLINE, TripDatabase, ClinicalTrials, CINAHL, and Cochrane up to January 2024 to identify full-text studies in English evaluating the effectiveness of GP involvement. A meta-analysis was also performed. Results: Of 1018 records, 15 studies were included, with an intermediate quality assessment. Studies originated from the United States (*n* = 9), Europe (5), Singapore (1), and China (1). Eight studies investigated educational programs for GPs, while seven focused on organizational or technological interventions to enhance immunization practices. Twelve studies reported increased vaccine uptake after intervention. Vaccines addressed included influenza, SARS-CoV-2, pneumococcal, zoster, and trivalent (diphtheria, tetanus, pertussis). Interventions involving GPs in PD vaccination campaigns, focusing on organizational or technological strategies, demonstrated a significant increase in vaccine uptake (OR = 1.15; 95% CI: 1.03–1.27; *p* < 0.0001; I^2^ = 96%). Conclusions: GPs emerged as valuable allies for PDs due to their extensive territorial reach and trusted relationships with patients. Additionally, up-to-date organizational and technological tools could play a decisive role in increasing vaccine uptakes. This study, offering valuable insights into the effectiveness of GPs involvement, may be useful to implement similar intervention in different contexts.

## 1. Introduction

Immunization stands as a cornerstone of global health, annually preventing millions of deaths by enhancing the body’s immune response and providing protection against more than 20 life-threatening diseases across all age groups [1]. The Advisory Committee on Immunization Practices (ACIP), developing recommendations on how to use vaccines, emphasizes that vaccination is one of the most crucial public health measures for preventing infectious diseases and that maintaining high vaccination coverage rates within the population is essential for controlling vaccine-preventable diseases that affect persons throughout their life span and decreasing associated morbidity and mortality [2]. A 2024 Lancet article by Andrew Shattock et al. pointed out that vaccinations have saved 154 million people from dying since 1974; vaccinations are also responsible for 40% of the decline in infant mortality globally [3]. Despite this, global vaccination coverage is still patchy and incomplete, with the WHO highlighting that 14.5 million children will still remain unvaccinated in 2023. The COVID-19 pandemic has further exacerbated this issue, contributing to a dramatic decline in vaccination coverage, which has not yet returned to the pre-pandemic levels seen in 2019 [4]. Even post-pandemic, concerns about vaccine safety, misinformation, and distrust in the healthcare system continue to drive vaccine hesitancy, further limiting vaccination coverage [5]. Due to these considerations, the Strategic Advisory Group of Experts on Immunization (SAGE), in their development of the Immunization Agenda 2030, emphasized that it will be essential for each country to establish catch-up vaccination policies and schedules, with healthcare workers adequately trained to screen and vaccinate. They also highlighted the importance of leveraging all available tools to promote vaccination and minimize missed opportunities, including integrating catch-up vaccination efforts within primary healthcare services [6].

As the WHO underlined, Primary Health Care (PHC) enabled health systems to meet an individual’s comprehensive health needs, including health promotion and disease prevention. PHC is widely regarded as the most inclusive, equitable, and cost-effective approach to achieving universal health coverage [7]. GP (GPs) is the key medical professional within the context of PHC. Through sustained relationships with individual patients and their families, GPs can establish a long-term therapeutic alliance grounded in trust and solidarity. The interpersonal GPs’ skills, as well as their ability to engage therapeutically with patients, are crucial to achieving positive clinical outcomes, as emphasized by the World Organization of Family Doctors (WONCA) [8]. A 2019 cross-over study by Christine Cohidon and colleagues emphasized the pivotal role of GPs within PHC in enhancing prevention practices. The study demonstrated how GPs, in collaboration with PDs, can adopt systematic, context-specific approaches to health promotion [9]. GPs could play a significant role in increasing immunization rates among adults, particularly the elderly, as these populations frequently consult their GP for the management of chronic conditions, as noted by A. Sessa et al. from the Italian College of GPs in an editorial paper in 2015 [10]. Furthermore, advice from one’s own GP has been shown to be a key determinant influencing vaccination acceptance, as suggested by a study conducted by Renske Eilers et al. in Dutch patients over 50 years of age [11].

PDs worldwide have involved GPs and PHC departments in numerous ways to develop vaccination campaigns, particularly during the COVID-19 pandemic. The European Centre for Disease Prevention and Control (ECDC) has produced a comprehensive report detailing all the strategies employed during the 2021 COVID-19 vaccination campaign [12]. These PDs engagement strategies have included interventions such as reminder/recall systems for vaccination appointments using messaging platforms, recommendations from GPs, or a combination of these approaches. The United States Centers for Disease Control and Prevention (CDC) has also compiled these methods in its field guide, which was designed to support the efforts of health departments and community organizations [13]. However, the effectiveness of these engagement strategies is not always easy to determine.

This paper aimed to analyze effective strategies for GP involvement in PD vaccination campaigns. Rather than focusing on a specific vaccination campaign or patient population, it aimed at finding the most effective approaches for enhancing vaccine uptake.

## 2. Materials and Methods

### 2.1. Search Strategy

This systematic review was performed in alignment with the PRISMA guidelines and the standards for reporting systematic reviews and meta-analyses [14]. The protocol for the systematic review was registered in the International Prospective Register of Systematic Reviews (ID: CRD42024503102). To address the primary research objective, a PICO (Population, Intervention, Comparator, Outcome) framework was formulated, through the following key targeted search terms: (P) GPs; (I) involvement of GPs in vaccination campaign; (C) none or usual care; (O) am increase or decrease in vaccine uptake and acceptance in GP’s patient population. A systematic literature search was conducted across MEDLINE (PubMed), Cochrane Central Register of Controlled Trials (CENTRAL), CINAHL (EBSCO), and PsycINFO (EBSCO) databases up to February 2024. The search aimed to identify all published articles evaluating effective strategies to involve GPs in vaccination campaigns, with a focus on research published within the past 10 years to capture recent advancements. The inclusion criteria were restricted to studies involving human participants, including randomized controlled trials (RCTs), Clinical Trials, Clinical Studies, and Observational Studies, with full-text availability. Search strategies were tailored to the requirements of each database using the following Boolean operators and relevant keywords: (GP OR “GPs” OR “family doctor * ” OR “General practitioner * ” OR “primary care practice * ” OR “primary care physician * ” OR “primary care doctor * ”) AND (“vaccin * ”)) (“7 February 2014” [Date-Entry]: “7 February 2024” [Date-Entry]. Finally, with a snowball technique, we examined references cited in the primary papers to identify additional eligible papers.

### 2.2. Inclusion and Exclusion Criteria

The inclusion criteria were defined as follows: (1) language: studies published in English; (2) study design: Randomized Controlled Trials, Clinical Trials, Clinical Studies, and Observational Studies presenting original primary data; (3) free full text available; (4) population of interest: GP; (5) intervention: involvement of GPs in vaccination campaign; (6) outcome measurement: vaccine uptake and acceptance in GP’s patient population; (7) comparison: not relevant, both studies with or without control groups were included.

The exclusion criteria were: (1) articles not pertinent to the research topic; (2) healthcare professionals different from GPs; (3) studies with interventions in pediatric patients; interventions aimed to investigate GPs’ knowledge toward vaccination; (4) study protocol or other papers without original data. Table 1 summarized the PICOS (Patients, Interventions, Comparators, Outcomes, and Study Designs) eligibility criteria.

### 2.3. Data Extraction and Quality Evaluation

Titles and abstracts were screened by reviewers to identify studies that satisfied the inclusion and exclusion criteria. Full texts of potentially eligible studies were retrieved after duplicate removal and independently assessed by six reviewers (AC, GM, FS, CC, DG, and MM) using a standardized, pre-tested data extraction form. Any disagreements regarding study eligibility were resolved through discussion within the research team. Data extracted from the study included: name of the first author, publication year, country, study design, care context, population study, type of intervention, type and numbers of outcome and results reported as vaccine uptake in target population. Data extraction followed the methods provided by the Cochrane Reviewers’ Handbook [15]. Study authors or investigators were contacted as needed to obtain additional information [16]. In accordance with PRISMA guidelines [14], the risk of bias for each included study was systematically evaluated. Three researchers (AC, GM, MM) independently and blindly assessed the studies using validated tools recommended for each study design. The “Version 2 of the Cochrane Risk-of-Bias Tool for Randomized Trials” (RoB-2) was employed to assess randomized controlled trials [17]. For observational studies, including cross-sectional and cohort designs, the “STROBE statement checklists” were utilized [18]. Quasi-experimental studies were evaluated using the “Risk of Bias in Non-Randomized Studies of Interventions” (ROBINS-I) tool [19]. These assessments ensured a consistent and rigorous approach to evaluating the methodological quality of the included studies. Disagreements among reviewers regarding quality scores were resolved through discussion. If consensus could not be reached, a fourth blinded reviewer (DG) acted as a tiebreaker. The risk-of-bias assessment focused on the primary outcome of interest: vaccine uptake and acceptance within the patient population of GPs. This methodological approach was conducted in alignment with PRISMA guidelines [14].

The RoB-2 tool assesses five domains of bias: (1) bias arising from the randomization process, (2) bias due to deviations from intended interventions, (3) bias resulting from missing outcome data, (4) bias in the measurement of outcomes, and (5) bias in the selection of reported results. Each domain is evaluated using signaling questions with predefined responses: yes, probably yes, probably no, no, or no information. These responses inform an overall risk-of-bias judgment, categorized as low risk, some concerns, or high risk. The STROBE (Strengthening the Reporting of Observational Studies in Epidemiology) statement, a 22-item checklist, is designed to improve reporting quality in observational studies, including cohort, cross-sectional, and case–control designs. Studies were graded based on prior research as follows: poor quality (0–14), intermediate quality (15–25), and good quality (26–33) [18]. The ROBINS-I (Risk of Bias in Non-Randomized Studies of Interventions) tool evaluates bias in nonrandomized studies that compare health outcomes across two or more interventions. It provides a structured evaluation of potential biases across various domains before and after intervention implementation. The ROBINS-I tool evaluates seven domains, offering a structured framework for assessing biases in nonrandomized studies examining intervention effects. The first two domains focus on issues present before the initiation of the interventions being compared, while the third domain addresses the classification of interventions. The remaining four domains examine post-intervention biases, including those arising from missing data, outcome measurement, and the selection of reported results. Response options for each domain mirrored those in RoB-2, with overall risk-of-bias ratings categorized as low, moderate, serious, critical, or no information [19].

After the descriptive analysis of the included studies, separate meta-analyses for the different interventions were performed. We assessed statistical heterogeneity to determine the appropriateness of combining studies for meta-analysis. Heterogeneity was evaluated using graphical forest plots and by calculating the I^2^ statistic, which quantifies the proportion of variance in effect estimates attributable to heterogeneity rather than sampling error (chance). An I^2^ value greater than 40% was considered indicative of substantial heterogeneity. Following the Cochrane Handbook for Systematic Reviews of Interventions [15], if fewer than five studies were included or substantial heterogeneity was observed, a random-effects model was applied using the DerSimonian and Laird method to compute random-effects estimates [20]. Forest plots were generated to present effect estimates with 95% confidence intervals. All data analyses were conducted using RevMan software (Version 5.3; The Nordic Cochrane Centre, The Cochrane Collaboration, Copenhagen, Denmark, 2014) [21].

## 3. Results

### 3.1. Selection and Characteristics of the Study

**Figure 1:**
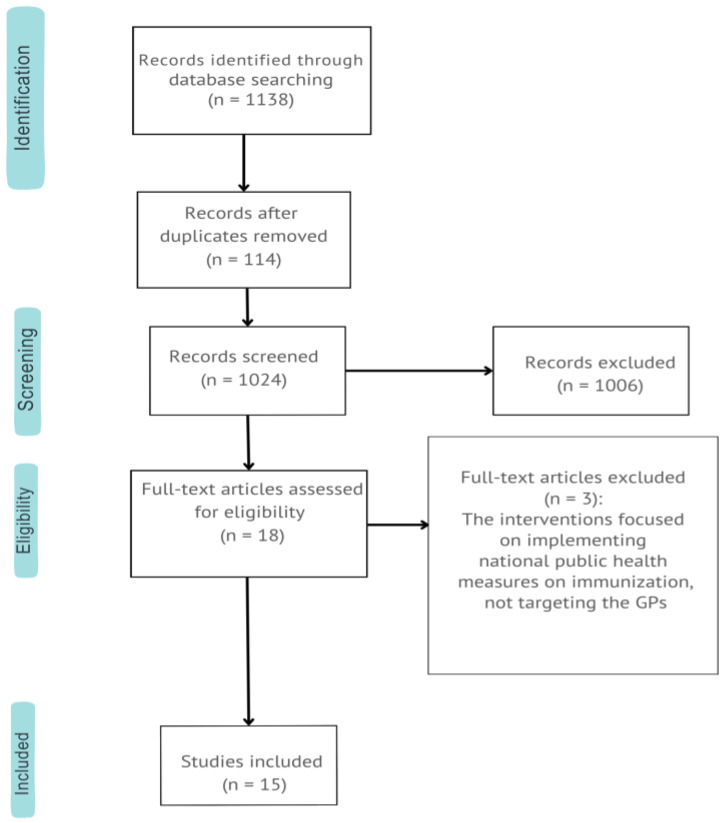
PRISMA flow diagram (PRISMA 2020 flow diagram for new systematic reviews which included searches of databases and registers only).

A total of 1138 articles were retrieved, with 1006 excluded during title and abstract screening due to misaligned focus (e.g., pediatric populations or GPs’ vaccination knowledge). Of 18 full-text articles reviewed, 15 met inclusion criteria (Table 1) and were analyzed, as shown in the PRISMA flow diagram (Figure 1). Exclusions primarily involved interventions targeting national public health measures rather than GPs. Included studies comprised two observational studies [22,23], two non-RCTs [24,25], and eleven RCTs [26,27,28,29,30,31,32,33,34,35,36].

### 3.2. Quality Assessment

The risk of bias was assessed using appropriate tools, as detailed in the Methods section. The ROBINS-I tool was applied to two quasi-experimental studies [22,23], both rated as having a moderate risk of bias due to participant selection and confounding factors. RCTs [24,25,26,27,28,29,30,31,32,33,34] were evaluated using the RoB-2 tool, with some, including studies by Emily Herrett [31], Richard K. Zimmerman [32], Chyongchiou J. Lin [33], and Mary Patricia Nowalk [34], identified as having ’some concerns’ related to randomization and intervention adherence. The remaining RCTs demonstrated a low risk of bias. Two observational studies [20,21], assessed using the STROBE checklist, were rated as intermediate quality. Table 2 summarizes the quality assessment findings.

### 3.3. Main Characteristics of the Included Studies

The geographic distribution of the included studies was as follows: USA (*n* = 9), Europe (*n* = 4), China (*n* = 1), and Singapore (*n* = 1). In fourteen cases, the studies were conducted in healthcare facilities, while only one study focused on individual GPs’ outpatient clinics. Eight interventions centered on providing information related to vaccination in general practice settings, whereas the remaining seven involved technological enhancements to standard immunization practices.

The vaccines targeted by these interventions included FLU vaccination (*n* = 7), SARS-CoV-2 vaccination (*n* = 3), pneumococcal vaccination (*n* = 1), and diphtheria-tetanus-pertussis (DTP) vaccination (*n* = 1), with three interventions addressing multiple vaccines. Twelve of the studies demonstrated an increase in vaccination uptake.

The study characteristics are summarized in Table 3. As previously mentioned, the interventions across the 15 included studies varied in design, yet all reported quantitative data on the measured outcome (vaccine uptake). To classify the types of interventions, we identified two main categories of GP involvement: the first category encompassed studies focused on implementing educational programs for GPs related to vaccination [22,23,24,26,30,34,35,36]; the second category involved interventions aimed at introducing organizational or technological enhancements in GPs’ vaccination practices [25,27,28,29,31,32,33].

#### 3.3.1. Efficacy of GPs Involvement in Vaccination Educational Program

The eight included studies explored diverse interventions, including observational cohorts, randomized controlled trials (RCTs), and quality improvement initiatives, implemented in various settings across the United States, China, and Norway. The programs assessed range from targeted training and decision-support tools to comprehensive practice transformation strategies, all aimed at increasing vaccination uptake for diseases such as COVID-19, FLU, and pneumococcal infections.

One of the studies, an observational cohort by Emily Gruber et al. [22], conducted in Maryland, USA, included 245,349 patients. The study aimed to compare COVID-19 vaccine uptake among patients of GPs participating in the Maryland Primary Care Program (MDPCP)—a model that integrates primary care with public health through expanded care management, integrated behavioral health, data-informed care, and screenings for social needs and referrals—against patients of GPs not involved in the program. The MDPCP group achieved a full vaccination rate of 84.47%, compared to 77.93% in the non-participating group, reflecting a 6.5 percentage point difference (*p* < 0.001).

A 2023 Chinese RCT by Yating You et al. [26] involved 3814 elderly individuals over 60 years old across 24 Community Health Centers (CHCs). This study evaluated the effectiveness of GP recommendations on FLU vaccination uptake. In the intervention group, 2457 patients were vaccinated, representing 1100 more vaccinations during the 2017–2018 flu season compared to 1493 patients in the control group, who had 86 fewer vaccinations compared to the 2016–2017 season.

Two additional studies focused on multi-component interventions. An American non-RCT by Natalia Y. Loskutova et al. [24] assessed 23 providers from the Academy of Family Physicians National Research Network, while a Norwegian RCT by Marit Tuv et al. [30] evaluated 25 GPs across 11 medical centers. The U.S. study assessed the effectiveness of Clinical Decision Support (CDS) algorithms for improving immunizations for FLU (FLU), pneumococcal conjugate vaccine (PCV), and herpes zoster (HZ) based on current vaccination guidelines. The Norwegian study examined the effectiveness of GP-specific training on COVID-19 vaccination and managing vaccine hesitancy. In both studies, control groups were GPs in similar contexts who did not receive the educational intervention. The U.S. study reported significant increases in vaccination rates after 12 months for FLU (Intervention by 6.9 percentage points, *p* = 0.001; control by 6.2 percentage points, *p* = 0.01), PCV (Intervention by 18.6 percentage points, *p* < 0.0001; control by 16.7 percentage points, *p* < 0.0001), and HZ (Intervention by 4.8 percentage points, *p* < 0.0001; control by 7.3 percentage points, *p* = 0.001). The Norwegian study found that 8.9% (*n* = 18/202) of the intervention group and 5.3% (*n* = 24/452) of the control group were vaccinated for COVID-19 (OR 1.72; 95% CI = 0.90 to 3.28).

A 2020 observational cohort study by Steven Kawczak et al. [23] evaluated a three-stage quality improvement initiative combined with continuing medical education (CME) to improve FLU and PCV vaccination rates among 273 primary care physicians in the Cleveland Clinic Quality Alliance network. The intervention included: Stage A—baseline assessment, Stage B—learning interventions with action planning, and Stage C—reassessment. Data from a control group of non-participating clinicians were also analyzed. Among patients aged ≥65 years in the intervention group, FLU vaccination rates increased significantly from 56.2% at Stage A to 58.7% at Stage C (*p* < 0.001), while rates for high-risk patients aged 18–64 rose from 38.6% to 40.4% (*p* < 0.001). PCV vaccination rates also improved, increasing in the intervention group from 80.6% to 82.7% (*p* < 0.001) and in the control group from 56.7% to 58.2% (*p* < 0.001). For high-risk adults, PCV vaccination rates rose from 40.4% to 43.8% in the intervention group (*p* < 0.001) and from 28.5% to 30.5% in the control group (*p* < 0.001).

Three U.S.-based crossover randomized controlled trials (RCTs) by Richard K. Zimmerman et al. [34], Chyongchiou J. Lin et al. [35], and Mary Patricia Nowalk et al. [36] examined the effectiveness of the 4 Pillars™ Practice Transformation Program, also referred to as the 4 Pillars™ Immunization Toolkit, in 25 primary care practices across Houston and Pittsburgh. This program aims to address vaccine-preventable diseases by identifying vaccination barriers and implementing tailored strategies to overcome them. At the conclusion of Year 1, practices could choose to extend the intervention into Year 2; four practices elected to do so. In Year 2, the Year 1 control sites initiated the intervention, while the four Pittsburgh practices that continued the program were merged with the former control sites to form the active intervention group. The study assessed the intervention’s effectiveness on vaccine uptake for pneumococcal polysaccharide vaccine (PPSV), pneumococcal conjugate vaccine (PCV), FLU (FLU) vaccine, and tetanus–diphtheria toxoids and acellular pertussis vaccine (Tdap) in a total of 70,549 patients. At the end of the study, PPSV rates at individual sites ranged from 43.4% to 94.7%. Both active intervention and maintenance groups showed significant improvements in PPSV rates from Year 1 to Year 2 (*p* < 0.001). By Year 2, 79% of practices (19/24) had PPSV rates of 70% or higher, and 58% (14/24) had rates of 80% or higher. PCV rates increased significantly more in the active intervention sites compared to maintenance sites (*p* < 0.001 for Pittsburgh and *p* < 0.01 for Houston). For FLU vaccination, both intervention and maintenance groups significantly increased vaccination rates and reduced missed opportunities. In Pittsburgh, the percentage point change in FLU vaccination did not differ between intervention (1.44 PP) and maintenance (1.4 PP) groups, but in Houston, the intervention group showed a significantly higher increase (3.6 PP) compared to the maintenance group (1.7 PP; *p* < 0.001). For Tdap, both cities saw a greater increase in vaccination rates in the intervention groups (6.2 PP) compared to maintenance groups (2.2 PP in Pittsburgh and 4.1 PP in Houston; *p* < 0.001).

**Figure 2:**
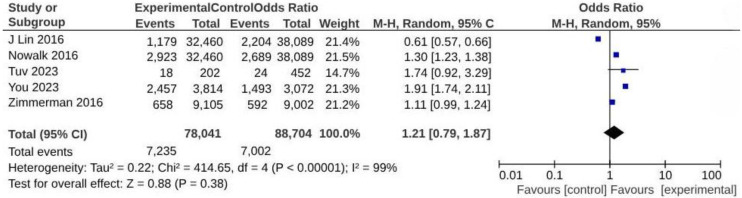
Meta-analysis assessing vaccine uptake from interventions involving GP in vaccination educational program. Studies are presented in alphabetical order [26,30,34,35,36].

Based on the methodological nature of the studies included in the review or the lack of available data, only five studies were eligible for the meta-analysis [26,30,34,35,36]. The analysis demonstrated no significant increase in vaccine uptake associated with the involvement of general practitioners (GPs) in vaccination educational programs (OR = 1.21; 95% CI: 0.79–1.87; *p* < 0.00001; I^2^ = 99%) (Figure 2).

#### 3.3.2. Efficacy of GPs’ Involvement in Organizational or Technological Implementations

The seven remaining included studies explored innovative organizational and technological strategies aimed at improving immunization practices among GPs. These interventions, ranging from culturally tailored outreach and reminder systems to the use of informational materials and text message campaigns, targeted diverse populations in various healthcare settings. Even these studies assessed their effectiveness in enhancing vaccination uptake for FLU, COVID-19, and pneumococcal diseases, offering valuable insights into the potential of structured and technology-supported approaches in overcoming barriers to immunization.

A 2022 randomized controlled trial (RCT) by Tracy A. Lieu et al. [27] was conducted within the Permanente Medical Group (TPMG) of Kaiser Permanente Northern California (KPNC) and included 8287 Latino and Black individuals aged 65 years and older. Participants who had not received a COVID-19 vaccination despite previous outreach were randomly assigned to one of three groups: (1) outreach via electronic secure messages and/or mail from their general practitioners (GPs; standard GP group), (2) outreach incorporating culturally tailored content (culturally tailored group), or (3) usual care, which involved no outreach messages (usual care group). Vaccination rates were significantly higher in the culturally tailored group compared to the usual care group (adjusted hazard ratio [aHR], 1.22; 95% CI, 1.09–1.37; *p* < 0.001) and in the standard GP group compared to usual care (aHR, 1.17; 95% CI, 1.04–1.31; *p* = 0.007). However, the difference between culturally tailored outreach and standard GP outreach was not statistically significant (aHR, 1.04; 95% CI, 0.94–1.17; *p* = 0.42).

Two RCTs by Peter G. Szilagyi et al. [28,29], conducted in 2020 and 2021 at UCLA Health System practices, assessed strategies for improving FLU (FLU) vaccination. The first trial involved 164,205 patients randomized into four groups: no reminders, one, two, or three reminders sent through the Epic™ EHR patient portal. Vaccination rates were significantly higher in the reminder groups (32.8%) compared to the control (32.0%; *p* = 0.001). The second trial, involving 196,486 patients, tested six messaging strategies, including pre-commitment letters and gain- or loss-framed reminders. This study found no meaningful differences in vaccination rates between groups, including those receiving pre-commitment messages (e.g., 36.5% without vs. 37.0% with, *p* > 0.05) or various message framings.

A 2023 French non-RCT by Laurent Rigal et al. [25] involved 14 GPs across three multi-professional health centers. The study enrolled 810 adults listed with participating GPs who were eligible for the 2019–2020 FLU vaccination campaign and unvaccinated as of 2 January 2020 (mid-campaign). GPs in the intervention group sent a standardized letter inviting eligible, unvaccinated patients to receive the FLU vaccine, while GPs in the control group continued with standard clinical practice. At the end of the campaign, vaccination coverage in the intervention group was 14.7% (95% CI [11.6%, 17.9%]) compared to 1.7% (95% CI [1.0%, 4.3%]) in the control group, resulting in a 13.1 percentage point difference between the two groups (*p* < 0.001).

Two additional studies focused on informational materials to promote vaccination. A 2019 crossover RCT by Hanley J. Ho et al. [31] in Singapore involved 22 GP clinics using flyers and posters for a 3-month intervention period. This resulted in higher vaccination rates for FLU (5.9% vs. 4.8%; *p* = 0.047) and pneumococcal disease (PCV) (5.7% vs. 3.7%; *p* = 0.001) compared to a control period. In contrast, a 2019 RCT by Christophe Berkhout et al. [32] in 75 French GP waiting rooms found no significant impact of additional FLU vaccination posters and pamphlets on vaccination uptake (Relative Risk = 1.01; 95% CI, 0.97–1.05; *p* = 0.561).

Finally, an observational study by Emily Herrett et al. [33] examined text messaging reminders for FLU vaccination in 156 American general practices involving 102,257 at-risk patients aged 18–64. Practices using text reminders (N = 77) achieved slightly higher vaccination uptake (52.4%) compared to standard care (50.7%; OR 1.11; 95% CI, 1.00–1.25).

**Figure 3:**
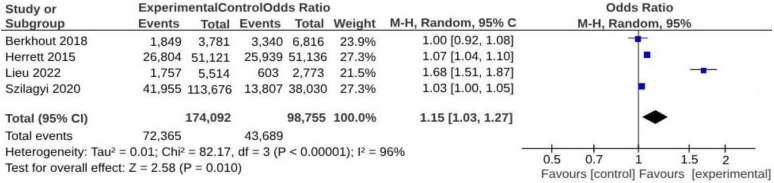
Meta-analysis assessing vaccine uptake from interventions involving GPs in organizational or technological implementations. Studies are presented in alphabetical order [27,28,32,33].

Due to the methodological nature of the studies included in the review or the lack of available data, only 4 studies were eligible for inclusion in the meta-analysis [27,28,32,33]. The analysis revealed a significant increase in vaccine uptake associated with interventions designed to engage GPs in organizational or technological implementations (OR = 1.15; 95% CI: 1.03–1.27; *p* < 0.0001; I^2^ = 96%) (Figure 3).

## 4. Discussion

This systematic review with meta-analyses assessed the effectiveness of GPs’ involvement in vaccination campaigns conducted by PDs internationally, emphasizing both educational aspects and the implementation of organizational and technological innovations. The review not only described the impact of these interventions on vaccine uptake but also examined the nature of the implementations compared to standard GP vaccination practices.

Among the 15 studies selected, 9 were from the United States, 4 from Europe, and 2 from Asia. The global prevalence of GP involvement in PD vaccination campaigns suggested their potentially crucial role in addressing vaccine hesitancy. This hypothesis may be supported by a 2023 cross-sectional study by Kemmyo Sugiyama et al. in Japan, which found that being under the care of a GP was associated with an increased likelihood of COVID-19 vaccination [37]. Similarly, in a 2017 observational study in Singapore involving 3700 individuals, over 50 identified having a GP as a positive predictor of flu vaccine uptake [38].

Fourteen of the fifteen studies included were conducted in multidisciplinary clinical settings with multiple GPs, suggesting a well-established international trend towards implementing community-oriented primary care (COPC) models. COPC focuses on healthcare as a relational process, often reducing health inequalities related to socioeconomic, structural, and environmental factors [39]. Reviews, such as the 2022 study by Chris G. Buse et al., have highlighted how the COPC approach can enhance integration and collaboration between primary care, public health, and preventive medicine, especially in rural and complex community settings [40]. This aspect was also supported by a recent Italian paper from 2024 conducted on 15,272 Italian citizens, which showed that territorial context variables such as altitude, urban planning, and the presence of a vaccination center could impact vaccine uptake [41]. Furthermore, logistical accessibility to healthcare facilities is identified as a significant factor influencing vaccination willingness among older adults, as noted in a systematic review by R. Eilers et al. of 1001 studies [42].

While the involvement of GPs in vaccination educational programs did not significantly increase vaccine uptake, their involvement in organizational or technological implementations was effective, with an odds ratio of 1.15 (95% CI: 1.03–1.27; *p* < 0.00001; I^2^ = 96%). These findings are supported by a systematic review conducted by Odone et al., which highlights the potential to enhance vaccine uptake and coverage through programs and interventions incorporating technological tools [43].

Technological implementations could play a crucial role in integrating primary prevention practices, such as vaccination, into primary care settings, as evidenced by a 2024 cohort retrospective study conducted on 1039 patients, where the vaccination reminder message served as the primary source of information by which patients became aware of the catch-up campaign [44]. Although digital systems are currently used in prevention practices, they are predominantly applied in tertiary prevention, as highlighted by a 2022 U.S. review [45]. Nevertheless, their broader application in the context of primary prevention could also prove to be cost-saving or cost-effective, as indicated in a 2023 systematic review by Wang et al., which screened 6860 studies [46].

This systematic review has several limitations. First, it included only studies published in English, potentially excluding valuable research published in other languages and introducing a risk of language bias. Second, gray literature was not considered, which may have resulted in the omission of relevant but unpublished or non-peer-reviewed studies. Third, we acknowledge that three studies (20% of the total) were conducted at the same sites, though the interventions were tailored to different vaccines. While we considered the potential for site-specific biases, our assessment of the methodological quality of each study revealed no significant factors that would unduly influence the results. However, we recognize that the inclusion of multiple studies from the same sites may limit the generalizability of these findings. Fourth, the substantial variation in GP roles across different countries and healthcare systems may limit the generalizability of these conclusions. Lastly, only seven of the included studies were rated as having a low risk of bias, which could impact the robustness of the findings. These factors should be taken into account when interpreting the results.

## 5. Conclusions

This systematic review examined the effectiveness of general practitioners’ involvement in vaccination campaigns within the prevention department. The included studies, consisting of RCTs, observational, and quasi-experimental designs, generally demonstrated medium to high quality and described various methods of GP engagement and outcomes across diverse global settings. While the adoption of purely educational interventions involving GPs did not show statistically significant increases in vaccine uptake, the meta-analysis results indicated that interventions targeting GPs to engage the adult population, with a focus on organizational and technological implementation, were effective. These findings highlight the potential of GPs as valuable partners in public health efforts to achieve vaccination targets. However, the need for more robust scientific evidence in this area remains, particularly in light of the increasing diversity of vaccination delivery settings. Potential avenues for future research include examining the impact of vaccination delivery sites on vaccine uptake, such as comparing practices that administer vaccines on-site to those that do not. Additionally, exploring the contributions of other healthcare professionals, such as pharmacists and nurses, in vaccine delivery could provide valuable insights for future research.

## Figures and Tables

**Table 1:** PICOS inclusion and exclusion criteria.

Parameter	Inclusion Criteria	Exclusion Criteria
Population	GP or other related professionals performing the same profession; e.g Primary care physician (PCP) or physicians operating in a Primary care center.	Healthcare professionals different from GPs (or other related professionals performing the same profession).
Intervention	Involvement of GPs in vaccination campaign	Interventions in pediatric patients, interventions aimed to investigate GPs’ knowledge toward vaccination
Comparator	None	None
Outcome	Vaccine uptake and acceptance in GP’s patient population	Another outcome
Study design	Experimental, quasi-experimental or observational study with original primary data and full-text studies written in English	Study Protocol or other papers not presenting original data (e.g., reviews, letters to editors, trial registrations, proposals for protocols, editorials, book chapters, conference abstracts).

**Table 2:** Quality assessment of RCTs, quasi-experimental, and observational studies.

Authors	Study Design	Tool for Assessment	Risk of Bias
Emily Gruber et al. [22]	Observational	STROBE	(19/33) Intermediate
Steven Kawczak et al. [23]	Observational	STROBE	(24/33) Intermediate
Natalia Y. Loskutova et al. [24]	Quasi-experimental	ROBINS-1	Moderate
Laurent Rigal et al. [25]	Quasi-experimental	ROBINS-1	Moderate
Yating You et al.[26]	RCT	Cochrane ROB Tool	Low
Tracy A. Lieu et al. [27]	RCT	Cochrane ROB Tool	Low
Peter G. Szilagyi et al. [28]	RCT	Cochrane ROB Tool	Low
Peter G. Szilagyi et al. [29]	RCT	Cochrane ROB Tool	Low
Marit Tuv et al. [30]	RCT	Cochrane ROB Tool	Low
Hanley J Ho et al. [31]	RCT	Cochrane ROB Tool	Low
Christophe Berkhout et al. [32]	RCT	Cochrane ROB Tool	Low
Emily Herrett et al. [33]	RCT	Cochrane ROB Tool	Some concerns
Richard K Zimmerman et al. [34]	RCT	Cochrane ROB Tool	Some concerns
Chyongchiou J Lin et al. [35]	RCT	Cochrane ROB Tool	Some concerns
Mary Patricia Nowalk et al. [36]	RCT	Cochrane ROB Tool	Some concerns

**Table 3:** Studies included in the review.

Author, and Publication Year	Study Design	Country	Care Context	Population	Intervention	Outcome	Result
Emily Gruber et al. 6 January 2023 [22]	Observational cohort study	USA Maryland	MDPCP practices (over 500 practices) and a matched cohort of other primary care practices not participating to the MDPCP	Total N: 245,349. N: 208,146 beneficiaries in the MDPCP group N: 37,203 beneficiaries in the non-MDPCP.	The MDPCP assisted in the COVID-19 response through four main initiatives: (1) offering data tools for targeted outreach, such as a real-time vaccine tracking system developed by CRISP; (2) facilitating early and coordinated distribution of resources, including vaccine allocation; (3) ensuring consistent communication between the MDH and practices; and (4) providing non-visit-based financial support to MDPCP practices.	Vaccination rates.	The MDPCP group was shown to have associations of higher uptake of COVID-19 vaccination, compared with the nonparticipating group. While 84.47% of the MDPCP group was fully vaccinated, 77.93% of the nonparticipating group was fully vaccinated (6.5—percentage point difference; *p* < 0.001).
Yating You et al. 17 January 2023 [26]	Randomized Controlled Trial	China Shenzhen	In China, CHCs a total of 24 health centers in 4 districts were selected, among which half were assigned to the intervention group and the other half to the control group	Total N: 6886 Intervention group: N: 3814 Control group: N: 3072	PCPs working in the intervention health centers recommend FLU vaccination to their patients who were aged 60 and above. PCPs working in the control CHCs did not provide FLU vaccination recommendations for their patients.	Changes in the number of older patients vaccinated in the 24 studied health centers during the 2017–2018 flu season compared to the 2016–2017 vaccination campaign.	In the intervention group, 2457 patients received the FLU vaccine, marking an increase of 1100 patients compared to the 2016–2017 flu season. In contrast, the control group saw 1493 patients vaccinated, a decrease of 86 compared to the baseline 2016–2017 flu season.
Tracy A. Lieu et al. 17 June 2022 [27]	Randomized Clinical Trial	USA California	GPs from TPMG, working in medical centers	Total N: 8287 Latino and Black individuals aged 65 years and older from 4 KPNC services. This patients were randomly allocated in one of these groups: N: 2767 individuals in the culturally tailored PCP outreach group; N: 2747 individuals in the standard PCP outreach group; N: 2773 individuals in the usual care group;	Unvaccinated individuals were randomized into three groups: standard PCP outreach (secure message or letter), culturally tailored outreach (addressing additional concerns like cost, immigration status, and racial disparities), and usual care (no outreach). Messages, sent in the PCP’s name, emphasized vaccine trust, safety, side effects, and appointment booking. After four weeks, unvaccinated individuals in the outreach groups received a follow-up postcard, while the usual care group received no outreach.	Time to receipt of COVID-19 vaccination within 8 weeks after initial study outreach.	At 8 weeks post-intervention, vaccination rates were 24.0% (664 individuals) in the culturally tailored PCP outreach group, 23.1% (635 individuals) in the standard PCP outreach group, and 21.7% (603 individuals) in the usual care group. Culturally tailored PCP outreach significantly increased vaccination rates compared to usual care (adjusted hazard ratio [aHR], 1.22; 95% CI, 1.09–1.37; *p* < 0.001), as did standard PCP outreach (aHR, 1.17; 95% CI, 1.04–1.31; *p* = 0.007). There was no significant difference between culturally tailored and standard PCP outreach (aHR, 1.04; 95% CI, 0.94–1.17; *p* = 0.42).
Peter G. Szilagyi et al. 18 May 2020 [28]	Randomized Clinical Trial	USA California	All 52 UCLA Health System primary care practices: 38 internal medicine, 5 medicine and pediatrics, 3 family medicine, and 6 pediatrics.	The sample included N: 164,205 active portal users, aged 6 months or older and eligible for FLU vaccination, who were primary care patients within the UCLA Health System and were randomly selected from the initial pool.	The study was a 4-arm, pragmatic, intention-to-treat randomized clinical trial involving 164,205 primary care patients assigned to one of four groups: no reminder (*n* = 41,070), 1 reminder (*n* = 41,055), 2 reminders (*n* = 41,046), or 3 reminders (*n* = 41,034). In the reminder groups, messages were sent via the patient portal, prompting patients with a secure email or text notification to log in and read a “message from your doctor,” without mentioning FLU vaccination in the title.	Receipt of one or more FLU vaccines, as documented in the electronic health record and supplemented with external data (e.g., pharmacies).	In the primary analysis FLU vaccination rates were 37.5% for those receiving no reminders, 38.0% for those receiving 1 reminder (*p* = 0.008 vs. no reminder), 38.2% for those receiving 2 reminders (*p* = 0.03 vs. no reminder), and 38.2% for those receiving 3 reminders (*p* = 0.02 vs. no reminder)
Peter G Szilagyi et al. 1 September 2021 [29]	Randomized Clinical Trial 6-arm RCT	USA California	All 53 internal medicine, medicine-pediatric, and family medicine primary care practices at UCLA.	The study included adult patients who used the Epic™ EHR patient portal within 12 months, stratified as follows: young adults aged 18–64 years without diabetes (N = 145,166), older adults aged 65 years and older without diabetes (N = 29,795), and adults aged 18 years and older with diabetes (N = 21,525). Non-active portal users were excluded.	A total of 196,486 patients, including young adults (N = 145,166), older adults (N = 29,795), and patients with diabetes (N = 21,525), were randomized into six groups (6-arm RCT): (1) control (no messages), (2) pre-commitment letter only, (3) pre-commitment letter plus loss-framed reminders, (4) pre-commitment letter plus gain-framed reminders, (5) loss-framed reminders only, or (6) gain-framed reminders only. Pre-commitment groups received a message in mid-October, while loss- and gain-framed groups received up to three portal reminders from late October to December if no FLU vaccination was recorded.	FLU vaccination rates between 10 January 2019 and 31 March 2020	FLU vaccination rates were low: 37% in young adults, 55% in older adults, and 60% in patients with diabetes. There were no significant differences in vaccination rates across pre-commitment or message framing (loss vs. gain) within any group. Both unadjusted and adjusted risk ratios showed no statistically or clinically significant impact of pre-commitment or message framing on vaccination rates.
Natalia Y. Loskutova 29 February 2020 [24]	Non-Randomized Clinical Trial (prospective intervention)	USA North Carolina	Ten sites 43 providers within the same organization (AFPNRN) were included; 23 primary care providers in the intervention arm of the study while 20 providers were in the comparator group	This study included patients aged 18 and older who received services from participating providers during 2013–2015 and were eligible for vaccinations. Eligibility included pneumococcal vaccination for those aged 65 and older or 19–64 with risk factors, FLU vaccination for all aged 18 and older, and zoster vaccination for those aged 60 and older.	The following components were provided exclusively to providers in the intervention group: standing orders, provider audit and feedback on vaccination rates, improved documentation of patients’ medical history, enhanced provider education on vaccines and patient communication, and increased patient awareness and acceptance of recommended vaccines through educational materials.	Effects of intervention on vaccination rates	Vaccination rates increased after 12 months in both intervention and comparator groups as follows: FLU: intervention by 6.9 percentage points (*p* = 0.001) and control by 6.2 percentage points (*p* = 0.01); pneumococcal vaccinations in older adults: intervention by 18.6 percentage points (*p* < 0.0001) and control by 16.7 percentage points (*p* < 0.0001); zoster: intervention by 4.8 percentage points (*p* < 0.0001) and control by 7.3 percentage points (*p* = 0.001); pneumococcal vaccinations in at-risk adults remained stable in the intervention group, while the comparator group increased by 3.8 percentage points (*p* = 0.003).
Marit Tuv 5 April 2023 [30]	Randomized Clinical Trial	Norway	Twenty-five GPs at 11 medical centers in Norway	Unvaccinated individuals over 18 years of age and at increased risk of severe COVID-19 were eligible. A total of 654 unvaccinated at-risk patients were identified: 202 were assigned to receive a phone call from their GP, while 452 were assigned to not receive a call.	Participants in the intervention group were contacted by their GPs via phone, while the control group received only standard care. During the calls, GPs explained that the purpose was to provide an opportunity to discuss and ask questions about the vaccine. GPs were given a one-page guide for conducting the phone call, along with a two-page document containing suggestions on how to address potential concerns raised by patients.	The proportion of participants registered as ’vaccinated against COVID-19′ in the Norwegian immunization Registry during the follow-up period was compared between the intervention and control groups.	The average follow-up period was 7.5 weeks. It is estimated that GPs successfully reached 76% (*n* = 154) of the patients they were assigned to call. At follow-up, 8.9% (*n* = 18/202) of the intervention group and 5.3% (*n* = 24/452) of the control group had been vaccinated (OR 1.72; 95% CI = 0.90 to 3.28).
Laurent Rigal 2023 [25]	Controlled non-randomized study	France	14 GPs in three multi-professional health centers	A total of 810 adults on a participating GP’s patient list were eligible for the 2019–2020 FLU vaccination campaign and were unvaccinated as of 2 January 2020, which was mid-campaign. Of these, 317 were assigned to the intervention arm, while 493 were assigned to the control arm.	On 2 January 2020, GPs in the intervention arm sent a standardized letter individually inviting their eligible patients not already vaccinated at mid-campaign to be vaccinated against FLU	FLU vaccination coverage estimated by the difference between the groups in their vaccination coverage at the end of the campaign (calculated from the NHIF databases)	At the end of the campaign, vaccination coverage was 14.7% (95% confidence interval [CI]: 11.6–17.9%) in the intervention group and 1.7% (95% CI: 1.0–4.3%) in the control group, resulting in a difference of 13.1 percentage points between the two groups (*p* < 0.001).
Hanley J Ho 2019 [31]	Pragmatic, cluster-randomized crossover trial	Singapore	22 private GP clinics in Singapore	The study included all patients aged 65 years or older, with or without chronic disease, who visited and were registered as clinic patients during the study period. In total, 8837 patients were considered. Of these, 4378 were included in the intervention periods, while 4459 were included in the control periods.	Clinics were assigned to a 3-month intervention period, which included a 1-month washout period, followed by a 4-month control period with usual care. The intervention materials consisted of informational flyers and posters with straightforward messages encouraging patients to receive FLU and pneumococcal vaccinations.	Differences in uptake rates for FLU and pneumococcal vaccinations between the intervention period and the control period.	Overall uptake rates were significantly higher in clinics during the intervention period compared with the control period for both FLU (5.9% vs. 4.8%; *p* = 0.047) and pneumococcal (5.7% vs. 3.7%; *p* = 0.001) vaccines.
Christophe Berkhout 2018 [32]	Randomized controlled trial	France	75 GPs’ waiting room	The study population consisted of patients aged 16 and older. The target group included patients over 65 years of age or those with chronic diseases requiring seasonal FLU vaccination, such as COPD or diabetes. In total, 3781 patients were included in the intervention periods, while 6816 patients were included in the control periods.	The study compared patient awareness between two settings: standard waiting rooms at 50 GPs’ offices (control group) and waiting rooms at 25 GPs’ offices where pamphlets and a poster on the FLU vaccine were provided, in addition to the standard mandatory information (intervention group).	Number of seasonal FLU vaccination units released in community pharmacies	No difference was observed in the number of FLU vaccination units delivered (Relative Risk = 1.01; 95% CI [0.97 to 1.05]; *p* = 0.561). However, having been vaccinated the previous year significantly increased the likelihood of revaccination (Relative Risk = 5.63; 95% CI [5.21 to 6.10]; *p* < 0.001).
Emily Herrett 2015 [33]	Cluster randomized trial	England	156 English primary care practices	The study involved 156 general practices that used text messaging software but had not previously used text message reminders for FLU vaccination. Eligible patients were aged 18–64 and classified as ’at-risk’. In total, 51,121 patients were included in the intervention periods, while 51,136 patients were included in the control periods.	Practices in the intervention arm (N: 77) were instructed to send text message reminders about FLU vaccination to their at-risk patients under 65. Practices in the standard care arm (N: 79) were asked to continue their FLU vaccination campaign as originally planned.	FLU vaccination rates uptake among patients aged 18–64 years in the seven prespecified risk groups during the period between 1 September and 31 December 2013.	In the standard care arm of the trial, mean vaccine uptake across practices was 50.7% and in the intervention, arm was 52.4% OR (95% CI) 1.11 (1.00 to 1.25).
Steven Kawczak 2020 [23]	Observational cohort study	USA	Primary care physicians employed by CCCAA and non-employed primary care physicians who are members of a regional Quality Alliance program	Out of 273 physicians from the Cleveland Clinic Quality Alliance network, 91.6% chose to participate in at least the first stage. Of these, 135 physicians (BMG [*n* = 8], CCF [*n* = 113], independent [*n* = 14]) progressed to Stage B (test group), while 100 physicians (BMG [*n* = 4], CCF [*n* = 87], independent [*n* = 9]) advanced to Stage C and completed the entire learning intervention.	The intervention was a three-stage quality improvement initiative incorporating CME learning activities. Stage A involved assessing practice to establish baseline performance. Stage B included participation in learning interventions and individualized action planning for practice change, while Stage C entailed reassessing practice. Data were also collected from a control group of clinicians who did not participate during the same period.	The rate at which patients of participating physicians received FLU and pneumococcal vaccines in accordance with guideline-based recommendations.	The intervention group showed significant increases in FLU vaccination rates, from 56.2% to 58.7% for patients aged ≥ 65 (*p* < 0.001) and from 38.6% to 40.4% for high-risk patients aged 18–64 (*p* < 0.001). Pneumococcal vaccination rates also increased, from 80.6% to 82.7% (*p* < 0.001) in the intervention group and from 56.7% to 58.2% (*p* < 0.001) in the control group for patients aged ≥ 65, with similar gains for high-risk adults in both groups.
Richard K Zimmerman 2016 [34]	Randomized controlled cluster trial CROSS-OVER RCT	USA	The study involved 25 primary care practices, stratified by city (Houston, Pittsburgh), location (rural, urban, suburban), and type (family medicine, internal medicine). In Pittsburgh, 19 clinics were enrolled, while 6 clinics participated in Houston.	GP’s patients aged 65 and older at baseline (N = 18,107; mean age 74.2; 60.7% female, 16.5% non-white, 15.7% Hispanic).	The 4PP, was implemented. At the end of Year 1, practices were given the option to continue the active intervention into Year 2, with four practices choosing to do so. Simultaneously, the Year 1 control sites began the intervention. For the Year 2 pre-post analyses, the four Pittsburgh practices that continued the intervention were combined with the Year 1 control sites and referred to as the active intervention group.	Vaccination rates for the 23-valent pneumococcal polysaccharide vaccine (PPSV) and pneumococcal conjugate vaccine (PCV), as well as percentage point (PP) changes in these rates. The primary outcomes were the cumulative PPSV and PCV vaccination rates reported at baseline, Year 1, and Year 2. Chi-square tests were conducted to assess differences in cumulative vaccination rates at various time points.	Cumulative PPSV vaccination rates for patients aged ≥ 65 increased significantly from baseline to Year 1 in both intervention and control groups, with gains of 6.5 to 8.7 percentage points (*p* < 0.001). Significant increases were observed at Houston sites (*p* < 0.001), but not Pittsburgh sites (*p* = 0.84). In Year 2, PPSV rates continued to improve, with 79% of practices achieving rates of at least 70% and 58% reaching 80%. Additionally, PCV rates increased significantly more in active intervention sites than in maintenance sites (*p* < 0.001 for Pittsburgh, *p* < 0.01 for Houston).
Chyongchiou J Lin 2016 [35]	Randomized controlled cluster trial CROSS-OVER RCT	USA	The study involved 25 primary care practices, stratified by city (Houston, Pittsburgh), location (rural, urban, suburban), and type (family medicine, internal medicine). In Pittsburgh, 19 clinics were enrolled, while 6 clinics participated in Houston.	GPs’ cohort of 70,549 adults seen in their respective practices (*n* = 24 with 1 drop out) at least once each year was followed. Baseline mean age was 55.1 years, 35% were men, 21% were non-white and 35% were Hispanic.	The 4PP was implemented. At the end of Year 1, practices had the option to continue the active intervention into Year 2, with four practices choosing to do so. Concurrently, the Year 1 control sites began the intervention. For the Year 2 pre-post analyses, the four Pittsburgh practices that continued in Year 2 were combined with the Year 1 control sites, creating the active intervention group.	FLU vaccination rate, was reported at the end of the baseline period (1 August 2012–31 January 2013) and the end of the intervention period (1 August 2013–31 January 2014) by site and intervention group for the Year 1 RCCT analyses.	After one year, both the intervention and control groups experienced significant increases in FLU vaccination rates, with improvements ranging from 2.7 to 6.5 percentage points (*p* < 0.001). Regression analyses indicated a higher likelihood of vaccination at sites with fewer missed opportunities (*p* < 0.001). After adjusting for missed opportunities, the intervention further increased vaccination rates in Houston (lower baseline rates) but not in Pittsburgh (higher baseline rates). During follow-up, vaccination likelihood improved at intervention sites and those that reduced missed opportunities (*p* < 0.005).
Mary Patricia Nowalk 2016 [36]	Randomized controlled cluster trial CROSS-OVER RCT	USA	The study involved 25 primary care practices, stratified by city (Houston, Pittsburgh), location (rural, urban, suburban), and type (family medicine, internal medicine). In Pittsburgh, 19 clinics were enrolled, while 6 clinics participated in Houston.	70,549 GPS’ patients ≥18 years who were seen in the practices ≥1 time each year, with a baseline mean age = 55 years; 35% were men; 56% were non-white; 35% were Hispanic and 20% were on Medicare	The 4PP was implemented. At the end of Year 1, practices could continue the active intervention into Year 2; four practices chose to do so. Meanwhile, the Year 1 control sites started the intervention. For the Year 2 pre-post analyses, the four Pittsburgh practices that continued the intervention were combined with the Year 1 control sites to form the active intervention group.	Cumulative Tdap vaccination rate reported at the end of baseline, Year 1 and Year 2	The baseline vaccination rate was 35%. In Year 1, Tdap vaccination rates increased more in the intervention groups (7.7 PP in Pittsburgh, 9.9 PP in Houston) than in the control groups (6.4 PP in Pittsburgh, 7.6 PP in Houston) (*p* < 0.001). In Year 2, active intervention groups had greater increases (6.2 PP) compared to maintenance groups (2.2 PP in Pittsburgh, 4.1 PP in Houston) (*p* < 0.001).

MDPCP: Maryland Primary Care Program; CHCs: Primarily Community Health Centers; TPMG: The Permanente Medical Group; UCLA: University of California, Los Angeles; AFPNRN: Academy of Family Physicians National Research Network; NHIF: national health insurance fund; CCCAA: Cleveland Clinic in Cleveland, Ohio and affiliated; 4PP: 4 Pillars™ Practice Transformation Program, also known as the 4 Pillars™ Immunization Toolkit (4pillarstoolkit.pitt.edu).

## Data Availability

Not applicable.
